# NONPARENTAL CAREGIVER DIFFERENCES IN LOCUS OF CONTROL AND GPA OF GRANDCHILDREN AND FOSTER CHILDREN

**DOI:** 10.1093/geroni/igad104.3066

**Published:** 2023-12-21

**Authors:** Maia McLin, Danielle Nadorff

**Affiliations:** Mississippi State University, Mississippi State, Mississippi, United States; Mississippi State University, Mississippi State, Mississippi, United States

## Abstract

There are approximately 5,950,690 children living with their grandparents, with about half being raised without a parent present. Children raised by non-parental caregivers are at risk of worse academic performance, and locus of control (LOC) is shown to relate to children’s academic performance. However, differences between caregiver types and this relation are not well known. The current study hypothesized that caregiver type would affect the strength of the association between LOC and academic performance in children raised by grandparents versus foster parents. Participants (323 caregiving grandparents (GP), m age = 55.66 yr; 105 foster parents (FP), m age = 34.45 yr) were recruited nationwide and surveyed via Qualtrics Panel Service. Measures included an adapted academic locus of control scale from the Child Trend’s Student Survey, and a question rating the degree to which the child receives “mostly As and Bs.” Controlling for age and socioeconomic status (SES) of caregiver, grandchildren had higher reported LOC, but did not differ in academic performance. Caregiver type was found to differentially affect the relation between LOC and academic performance, with a stronger association for foster children (GP r =.726; p < .001; FP r =.831; p < .001). A significant group difference was found (z =2.06; p < .05). These results suggest that kincare may buffer the impact of LOC on academic performance in children not raised by their parents. For those raised in foster care, it may be helpful to focus on increasing autonomy and sense of control regarding their grades.

